# Robotic-Assisted Laparoscopic Buccal Mucosal Graft Ureteroplasty and Ureteral Reimplantation for Repair of Complex Ureteral Strictures Using the Modular Carina™ System

**DOI:** 10.1590/S1677-5538.IBJU.2025.0660

**Published:** 2026-01-16

**Authors:** Wencong Han, Zhihua Li, Zhenyu Li, Guanpeng Han, Zheng Zhang, Kunlin Yang, Xuesong Li

**Affiliations:** 1 Peking University First Hospital Department of Urology Beijing China Department of Urology, Peking University First Hospital, Beijing, China; 2 Peking University Institution of Urology Beijing China Institution of Urology, Peking University, Beijing, China; 3 National Urological Cancer Center Beijing China National Urological Cancer Center, Beijing, China

## Abstract

**Purpose:**

Multifocal ureteral strictures pose significant challenges for reconstructive surgery due to their segmental distribution and the need to preserve the ureteral blood supply (1, 2). Robotic-assisted surgery, owing to its precision and minimally invasive advantages, has increasingly become a preferred approach (3). Although the da Vinci surgical system has long dominated this field, several novel robotic platforms have recently emerged with comparable safety and efficacy (4, 5). This study reports our experience with robotic-assisted laparoscopic buccal mucosal graft ureteroplasty combined with ureteral reimplantation for complex ureteral stricture repair using the modular Carina™ robotic surgical system.

**Materials and Methods:**

A 32-year-old man presented with a one-month history of flank pain and was found to have both proximal and distal ureteral strictures. Using the modular Carina™ robotic system, the procedure was performed as follows: dissection of the proximal stricture, longitudinal ureterotomy, posterior augmented anastomosis, harvesting of buccal mucosa for ventral onlay grafting; followed by dissection of the distal ureteral stricture and bladder wall and completion of a side-to-side ureterovesical anastomosis.

**Results:**

The procedure was completed successfully without conversion, with a total operative time of 272 minutes. The patient was discharged on postoperative day 7. Histopathological examination revealed granulomatous inflammation, and anti-tuberculosis therapy was initiated. The double-J stent and nephrostomy tube were removed 2 months postoperatively. During an 8-month follow-up, the patient's symptoms resolved, imaging demonstrated improvement of hydronephrosis, renal function remained stable, and no postoperative complications were observed.

**Conclusions:**

Robotic-assisted reconstructive surgery for complex ureteral strictures using the modular Carina™ robotic system is technically feasible. However, larger studies with longer follow-up are required to validate these preliminary findings.

**Available at:**VIDEO

## Data Availability

Uninformed
